# The draft genome and transcriptome of *Cannabis sativa*

**DOI:** 10.1186/gb-2011-12-10-r102

**Published:** 2011-10-20

**Authors:** Harm van Bakel, Jake M Stout, Atina G Cote, Carling M Tallon, Andrew G Sharpe, Timothy R Hughes, Jonathan E Page

**Affiliations:** 1 Banting and Best Department of Medical Research and Terrence Donnelly Centre for Cellular and Biomolecular Research, University of Toronto, 160 College St. Room 230, Toronto, ON, M5S 3E1, Canada; 2 Department of Biology, University of Saskatchewan, 112 Science Place, Saskatoon, SK, S7N 5E2 Canada; 3 National Research Council of Canada, Plant Biotechnology Institute, 110 Gymnasium Place, Saskatoon, SK, S7N 0W9, Canada; 4 Department of Molecular Genetics, University of Toronto, #4396 Medical Sciences Building, 1 King's College Circle, Toronto, ON, M5S 1A8 Canada

**Keywords:** Cannabaceae, cannabis, marijuana, hemp, genome, transcriptome, cannabinoid

## Abstract

**Background:**

*Cannabis sativa *has been cultivated throughout human history as a source of fiber, oil and food, and for its medicinal and intoxicating properties. Selective breeding has produced cannabis plants for specific uses, including high-potency marijuana strains and hemp cultivars for fiber and seed production. The molecular biology underlying cannabinoid biosynthesis and other traits of interest is largely unexplored.

**Results:**

We sequenced genomic DNA and RNA from the marijuana strain Purple Kush using shortread approaches. We report a draft haploid genome sequence of 534 Mb and a transcriptome of 30,000 genes. Comparison of the transcriptome of Purple Kush with that of the hemp cultivar 'Finola' revealed that many genes encoding proteins involved in cannabinoid and precursor pathways are more highly expressed in Purple Kush than in 'Finola'. The exclusive occurrence of Δ^9^-tetrahydrocannabinolic acid synthase in the Purple Kush transcriptome, and its replacement by cannabidiolic acid synthase in 'Finola', may explain why the psychoactive cannabinoid Δ^9^-tetrahydrocannabinol (THC) is produced in marijuana but not in hemp. Resequencing the hemp cultivars 'Finola' and 'USO-31' showed little difference in gene copy numbers of cannabinoid pathway enzymes. However, single nucleotide variant analysis uncovered a relatively high level of variation among four cannabis types, and supported a separation of marijuana and hemp.

**Conclusions:**

The availability of the *Cannabis sativa *genome enables the study of a multifunctional plant that occupies a unique role in human culture. Its availability will aid the development of therapeutic marijuana strains with tailored cannabinoid profiles and provide a basis for the breeding of hemp with improved agronomic characteristics.

## Background

One of the earliest domesticated plant species, *Cannabis sativa *L. (marijuana, hemp; Cannabaceae) has been used for millennia as a source of fibre, oil- and protein-rich achenes ("seeds") and for its medicinal and psychoactive properties. From its site of domestication in Central Asia, the cultivation of cannabis spread in ancient times throughout Asia and Europe and is now one of the most widely distributed cultivated plants [1]. Hemp fibre was used for textile production in China more than 6000 years BP (before present) [2]. Archaeological evidence for the medicinal or shamanistic use of cannabis has been found in a 2700-year old tomb in north-western China and a Judean tomb from 1700 years BP [3,4]. Currently cannabis and its derivatives such as hashish are the most widely consumed illicit drugs in the world [5]. Its use is also increasingly recognized in the treatment of a range of diseases such as multiple sclerosis and conditions with chronic pain [6,7]. In addition, hemp forms of cannabis are grown as an agricultural crop in many countries.

Cannabis is an erect annual herb with a dioecious breeding system, although monoecious plants exist. Wild and cultivated forms of cannabis are morphologically variable, resulting in confusion and controversy over the taxonomic organization of the genus (see [8] for review). Some authors have proposed a monotypic genus, *C. sativa*, while others have argued that *Cannabis *is composed of two species, *Cannabis sativa *and *Cannabis indica*, and some have included a third species, *Cannabis ruderalis*, in the genus. In light of the taxonomic uncertainty, we use *C. sativa *to describe the plants analyzed in this study.

The unique pharmacological properties of cannabis are due to the presence of cannabinoids, a group of more than 100 natural products that mainly accumulate in female flowers ("buds") [9,10]. Δ^9^-Tetrahydrocannabinol (THC) is the principle psychoactive cannabinoid and the compound responsible for the analgesic, antiemetic and appetite-stimulating effects of cannabis [11,12]. Non-psychoactive cannabinoids such as cannabidiol (CBD), cannabichromene (CBC) and Δ^9^-tetrahydrocannabivarin (THCV), which possess diverse pharmacological activities, are also present in some varieties or strains [13-15]. Cannabinoids are synthesized as carboxylic acids and upon heating or smoking decarboxylate to their neutral forms; for example, Δ^9^-tetrahydrocannabinolic acid (THCA) is converted to THC. Although cannabinoid biosynthesis is not understood at the biochemical or genetic level, several key enzymes have been identified including a candidate polyketide synthase and the two oxidocyclases, THCA synthase (*THCAS*) and cannabidiolic acid (CBDA) synthase, which form the major cannabinoid acids [16-18].

Cannabinoid content and composition is highly variable among cannabis plants. Those with a high-THCA/low-CBDA chemotype are termed marijuana, whereas those with a low-THCA/high-CBDA chemotype are termed hemp. There are large differences in the minor cannabinoid constituents within these basic chemotypes. Breeding of cannabis for use as a drug and medicine, as well as improved cultivation practices, has led to increased potency in the past several decades with median levels of THC in dried female flowers of ca. 11% by dry weight; levels in some plants exceed 23% [10,19]. This breeding effort, largely a covert activity by marijuana growers, has produced hundreds of strains that differ in cannabinoid and terpenoid composition, as well as appearance and growth characteristics. Patients report medical marijuana strains differ in their therapeutic effects, although evidence for this is anecdotal.

Cannabis has a diploid genome (*2n *= 20) with a karyotype composed of nine autosomes and a pair of sex chromosomes (X and Y). Female plants are homogametic (XX) and males heterogametic (XY) with sex determination controlled by an X-to-autosome balance system [20]. The estimated size of the haploid genome is 818 Mb for female plants and 843 Mb for male plants, owing to the larger size of the Y chromosome [21]. The genomic resources available for cannabis are mainly confined to transcriptome information: NCBI contains 12,907 ESTs and 23 unassembled RNA-Seq datasets of Illumina reads [22,23]. Neither a physical nor a genetic map of the cannabis genome is available.

Here, we report a draft genome and transcriptome sequence of *C. sativa *Purple Kush (PK), a marijuana strain that is widely used for its medicinal effects [24]. We compared the genome of PK with that of the hemp cultivars 'Finola' and 'USO-31', and the transcriptome of PK flowers with that of 'Finola' flowers. We found evidence for the selection of cannabis for medicinal and drug (marijuana) use in the up-regulation of cannabinoid 'pathway genes' and the exclusive presence of functional THCA synthase (*THCAS*) in the genome and transcriptome of PK.

## Results

### Sequencing the C. sativa PK genome and transcriptome

We obtained DNA and RNA samples from plants of PK, a clonally propagated marijuana strain that may have been bred in California and is reportedly derived from an "indica" genetic background [24]. Genomic DNA was isolated from PK leaves and used to create six 2 ×100-bp Illumina paired-end libraries with median insert sizes of approximately 200, 300, 350, 580 and 660 bp. Sequencing each of these libraries produced > 92 gigabase (Gb) of data after filtering of low-quality reads (see below), which is equivalent to approximately 110× coverage of the estimated ~820 Mb genome. To improve repeat resolution and scaffolding, we supplemented these data with four 2 × 44-bp Illumina mate-pair libraries with a median insert size of approximately 1.8 kb and two 2 × 44-bp libraries with a median insert size of approximately 4.6 kb, adding 16.3 Gb of sequencing data in 185 million unique mated reads. We also included eleven 454 mate-pair libraries with insert sizes ranging from 8 to 40 kb, obtaining > 1.9 Gb of raw sequence data (~2.3 × coverage of 820 Mb) and 2 M unique mated reads.

To characterize the cannabis transcriptome, we sequenced polyA+ RNA from a panel of six PK tissues (roots, stems, vegetative shoots, pre-flowers (i.e. primordia) and flowers (in early- and mid-stages of development)) obtaining > 18.8 Gb of sequence. To increase coverage of rare transcripts, we also sequenced a normalized cDNA library made from a mixture of the six RNA samples, obtaining an additional 33.9 Gb. The sequencing data obtained for the genomic and RNA-Seq libraries are summarized in Table 1.

**Table 1:** Purple Kush sequencing library statistics

Library	Insert size (bp)	**Raw No**.	Raw nt (Gb)	**Filtered No**.	Filtered nt (Gb)	% in ^a) ^assembly
**Genomic DNA, Illumina 2 × 100 bp paired-end reads**						
CS-PK_SIL-1a	181	143,951,601	28.0	124,499,863	23.8	82.2
CS-PK_SIL-1b	195	111,106,936	22.2	98,124,711	19.0	82.4
CS-PK_SIL-2a	313	93,774,355	18.8	81,421,333	15.3	84.7
CS-PK_SIL-3b	362	66,932,319	13.4	60,519,955	11.6	82.8
CS-PK_SIL-B	664	95,648,778	19.1	49,550,098	9.2	85.1
CS-PK_SIL-C	580	101,329,142	20.3	72,977,620	13.6	87.1
**Genomic DNA, Illumina 2 × 44 bp mate pair reads**						
CS-PK_2 kb-1a	1,926	36,057,086	3.2	24,688,690	2.2	75.0
CS-PK_2 kb-1b	1,846	32,385,628	2.8	24,405,458	2.1	76.8
CS-PK_2 kb-2a	1,850	37,761,064	3.3	29,927,921	2.6	75.5
CS-PK_2 kb-2b	1,787	37,111,622	3.3	28,744,604	2.5	77.1
CS-PK_5 kb-1	4,721	36,182,230	3.2	27,377,398	2.4	77.5
CS-PK_5 kb-2	4,585	64,613,144	5.7	50,712,974	4.4	79.9
**Genomic DNA, 454 mate pairs**						
CS-PK_8 kb-1	8,000	557,443	0.20	192,483	0.069	77.0
CS-PK_8 kb-2	8,000	484,033	0.17	176,405	0.063	74.5
CS-PK_8 kb-3	8,000	603,780	0.21	221,616	0.079	78.6
CS-PK_13 kb-1	13,000	430,642	0.11	96,503	0.030	75.1
CS-PK_20 kb-1	20,000	611,986	0.19	216,379	0.070	77.0
CS-PK_20 kb-2	20,000	575,618	0.21	228,811	0.081	77.0
CS-PK_30 kb-1	30,000	644,026	0.22	239,625	0.082	72.8
CS-PK_30 kb-2	30,000	536,273	0.15	150,510	0.048	73.4
CS-PK_40 kb-1	40,000	213,928	0.06	64,325	0.019	74.9
CS-PK_40 kb-2	40,000	627,945	0.21	241,189	0.079	76.8
CS-PK_40 kb-3	40,000	573,313	0.19	224,264	0.073	74.5
**RNA, Illumina 1 × 100 bp single-end reads**						
PK-Mid-flower	-	37,835,287	3.8	25,687,331	2.3	-
PK-Early-flower	-	37,472,665	3.7	25,434,724	2.3	-
PK-Pre-flower	-	54,026,640	5.4	35,522,980	3.2	-
PK-Shoot	-	55,653,984	5.6	36,204,828	3.3	-
PK-Stem	-	60,353,149	6.0	39,274,463	3.5	-
PK-Root	-	37,374,640	3.7	24,904,927	2.2	-
**RNA, Illumina 2 × 100 bp paired-end reads**						
PK-subtracted1	180	110,483,894	22.1	64,525,082	11.6	-
PK-subtracted2	180	82,190,044	16.4	46,291,148	8.3	-
PK-subtracted3	180	105,737,119	21.1	61,974,962	11.2	-
PK-subtracted4	180	48,599,953	9.7	26,505,457	4.8	-

a) Percentage of genomic DNA reads that could be mapped back to the canSat3 genome assembly

### Assembling the C. sativa PK genome and transcriptome

We used different approaches for the *de novo *assembly of the PK genome (SOAPdenovo [25]) and transcriptome (ABySS [26] and Inchworm [27]). To gauge the success of the outputs, and to refine the assemblies, we used both traditional measures (coverage, bases in assembly, N50, maximum contig size and contig count) as well as comparisons between the assembled versions of the genome and transcriptome.

For the transcriptome, we used two different assemblers, ABySS and Inchworm, to obtain the best possible coverage. Both assemblers were run on the individual tissue datasets and normalized cDNA libraries, as well as the full set of RNA-Seq data (summarized in Table 2). We used predicted splice junctions and the presence of apparent coding regions to orient the assembled transcripts and to perform quality control (QC). In general, Inchworm produced assemblies with a larger N50 than ABySS (Table 2); however, we also observed many cases in which adjacent transcripts (e.g. head-to-head transcripts that overlap in their termini) appeared to be merged. Therefore, we considered only Inchworm transcripts with a single blastx hit covering at least 70% of their length when merging assemblies. The filtered individual ABySS and Inchworm assemblies were combined by first selecting the largest transcript among sets of near-identical sequences from each assembly, followed by a second stage where transcripts with blunt overlaps were joined. This second step resulted in a significant improvement of transcript N50 from 1.65 to 1.80 kb (Table 2).

**Table 2:** Overview of transcriptome assembly stages

**Library**		**ABySS ^a)^**			**Inchworm ^a)^**	
		
	**N50 (kb)**	**Max (kb) ^b)^**	**Total (Mb) ^c)^**	**N50 (kb)**	**Max (kb) ^b)^**	**Total (Mb) ^c)^**
		
PK-Mid-flower	0.73	6.55	19.9	1.36	7.42	26.5
PK-Early-flower	0.64	4.94	16.7	1.06	6.11	24.0
PK-Pre-flower	0.80	6.74	21.4	1.56	7.89	28.0
PK-Shoot	0.69	6.16	20.2	1.34	7.41	26.8
PK-Stem	0.80	6.55	22.9	1.67	11.55	29.3
PK-Root	0.43	4.03	15.3	0.64	7.21	22.5
PK-tissue-all	0.62	8.74	26.7	-	-	-
PK-normalized1	-	-	-	1.78	10.96	31.1
PK-normalized2	-	-	-	1.72	7.89	34.2
PK-normalized3	-	-	-	1.84	8.19	34.7
PK-normalized4	-	-	-	1.71	7.10	32.1
PK-normalized-all	1.18	7.31	42.1	-	-	-
				
**Library**		**Combined**				
				
	N50 (kb)	Max (kb)	Total (Mb)			
				
Non-redundant	1.65	11.55	49.4			
Cap3 overlap merging	1.80	12.11	41.0			

a) Fields marked with '-' indicate library/assembly combinations that were not analyzed. Statistics are shown for transcripts that passed initial QC and that could be oriented according to coding strand.

b) Size of largest transcript in the assembly

c) Total number of non-gap bases in the assembly

**Figure 1:**
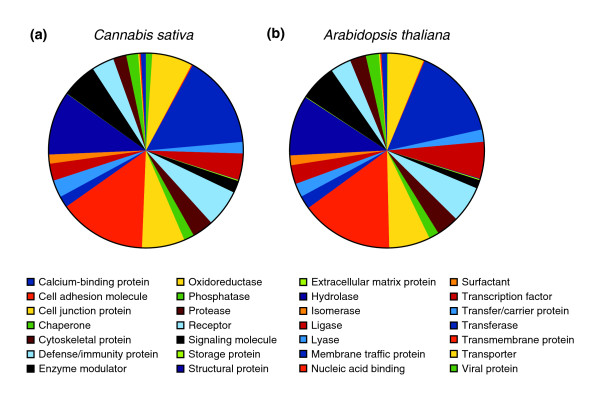
**Transcript classes in *Cannabis sativa *and *Arabidopsis thaliana***. Panther [28] was used to determine the distribution of transcripts in **(a) ***C. sativa *(PK) (30,074 representative transcripts) and **(b) ***A. thaliana *(31,684 transcripts). The high degree of similarity between both species indicates that all major functional classes are proportionally represented in the PK transcriptome assembly.

The final merged assembly contains 40,224 transcripts falling into 30,074 clusters of isoforms (Table 3). We selected the transcript with the largest open reading frame (ORF) as the representative for each cluster, resulting in a pruned assembly with an N50 of 1.91 kb. Most representative transcripts (83%) have a blastx hit in other plants, and the distribution of transcript classes, according to Panther [28], is nearly identical between PK and *Arabidopsis *(Figure 1), as is the total number of transcripts and the N50 (33,602 and 1.93 kb in *Arabidopsis*, respectively [29]). The total number of bases in representative *Arabidopsis *transcripts is, however, somewhat larger (50 Mb, [29]) which may indicate that some of the PK transcripts are partial or that genes are represented by more than one non-contiguous fragments. We noted a 3' end bias in the normalized cDNA library, presumably due to the polyA priming step (data not shown). Moreover, by combining near-identical transcripts during assembly merging and isoform clustering, we likely collapsed transcripts of large multi-copy gene families. Indeed, applying our isoform clustering algorithm to the *Arabidopsis *assembly reduces the total number of bases to 44 Mb, which is mostly due to the loss of transposable element genes. Overall, our assembled PK transcriptome is therefore very similar to the deeply characterized *Arabidopsis *transcriptome, both in size and composition.

**Table 3:** Genome and transcriptome assembly statistics

	Genome		Transcriptome	
		
	All ^a)^	With transcript ^a)^	All	Representative
Total bases (+ gaps)	786.6 Mb	532.3 Mb	40.63 Mb	33.20 Mb
Total bases (- gaps)	534.0 Mb	366.9 Mb	40.63 Mb	33.20 Mb
Scaffold N50	16.2 Kb	24.9 Kb	1.80 Kb	1.91 Kb
Number of scaffolds	136,290	45,776	40,224	30,074
Largest scaffold	565.9 Kb	565.9 Kb	12.11 Kb	12.11 Kb

^a) ^Only scaffolds and unplaced contigs larger than 400 bp are included in the genome assembly statistics.

**Figure 2:**
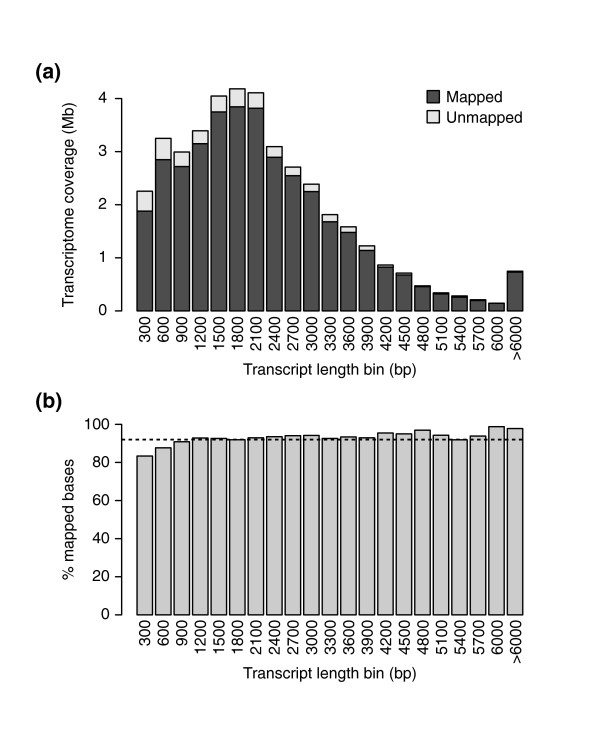
**Proportion of transcriptome mapping to genome assembly**. (**a**) A histogram showing the number of bases in the transcript assembly that could be mapped to the genome at 98% sequence identity, as a function of transcript length in 300 nt bins. (**b**) The proportion of transcriptome bases that could be mapped to the genome for the same bins as in (a). The black dashed line indicates the proportion of the transcriptome that is accounted for in the genome assembly.

Our genome assembly procedure first involved a series of filtering steps to remove low-quality reads, bacterial sequences (about 2% of all reads) and sequencing adapters. Mate-pair libraries (454 and Illumina) were further processed to remove duplicate pairs and unmated reads. We then assembled a small fraction of the Illumina data (1%) together with the 454 data, to reconstruct the mitochondrial (approximately 450 kb) and plastid (approximately 150 kb) genomes, and subsequently removed their highly abundant DNA sequences. The remaining reads were assembled with SOAPdenovo, resulting in a draft assembly that spans > 786 Mb of the cannabis genome and includes 534 million bp (Table 3). The Illumina mate-pair libraries had a significant impact on the assembly, increasing the N50 from 2 kb to 12 kb. Addition of the large-insert 454 data increased this to 16 kb (24.9 kb for scaffolds containing genes). Between 73% and 87% of the reads in each library could be mapped back to the draft genome (Table 1), indicating that our assembly accounts for most of the bases sequenced. As an additional measure of completeness, we also examined the proportion of the transcriptome represented in the genome assembly. Over 94% of assembled transcripts map to the draft genome over at least half of their length, and 83.9% of them are fully represented; that is, all bases of the transcript can be mapped to genomic contigs. Overall, 37.6 Mb (92.5%) of the complete transcriptome is accounted for in the genome assembly (Figure 2), and over 68.9% of transcripts are fully encompassed by a single scaffold. Thus, our draft genome assembly appears to represent a large majority of the genic, non-repetitive *C. sativa *genome.

The assembled *C. sativa *PK genome and transcriptome (canSat3) can be downloaded and browsed at a dedicated website [30]. The Cannabis Genome Browser combines the genome assembly with the transcriptome annotations, and has tracks for RNA-Seq data, single nucleotide variants (SNVs) and repeats.

### Expression of the cannabinoid pathway in C. sativa PK tissues

**Figure 3:**
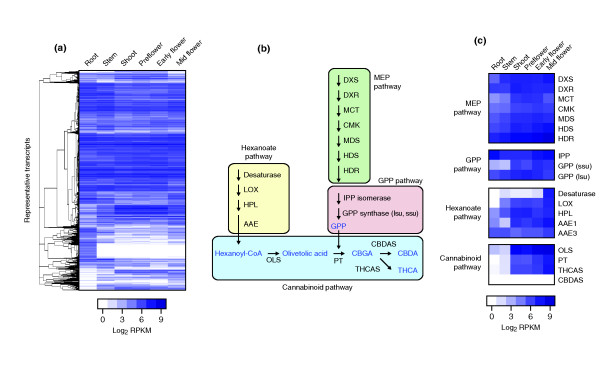
**Analysis of gene expression in PK tissues**. (**a**) RNA-Seq read counts for 30,074 representative transcripts (rows), expressed as log2 RPKM, were subjected to hierarchical agglomerative clustering based on their expression pattern across tissues (columns). (**b**) Schematic illustration of THCA and CBDA cannabinoid biosynthesis, including the production of fatty acid and isoprenoid precursors via the hexanoate, MEP and GPP pathways. Hexanoate could arise through fatty acid degradation, involving desaturase, lipoxygenase (LOX) and hydroperoxide lyase (HPL) steps. Activation of hexanoate by an acyl-activating enzyme (AAE) yields hexanoyl-CoA, which is the substrate for the polyketide synthase enzyme (OLS) that forms olivetolic acid. The prenyl side-chain originates in the MEP pathway, which provides substrates for GPP synthesis, and is added by an aromatic prenyltransferase (PT) [36]. The final steps are catalyzed by the oxidocyclases THCAS and CBDAS. Pathway enzymes and metabolic intermediates are indicated in black and blue, respectively. (**c**) Same data as (a), showing the expression levels for genes in the cannabinoid pathway and precursor pathways (rows) across the six assayed tissues (columns). The majority of the genes encoding cannabinoid and precursor pathway enzymes are most highly expressed in the flowering stages. Gene and pathway names correspond to those used in panel B.

Our first step in the functional analysis of the *C. sativa *genome was to examine the relative expression of each of the 30,074 representative transcripts in the six PK tissues, from which the RNA-Seq data were derived (Figure 3a). In metazoans (e.g. humans), different organs and tissues have different physiological functions, and consequently have unique gene expression profiles [31]. Therefore, many genes have a highly restricted expression pattern. By contrast, in plants, different photosynthetic tissues are often composed of a similar set of cell types. Moreover, photosynthetic processes and primary metabolic pathways have widespread expression, and only a minor proportion of transcripts appear to be uniquely expressed in a given cell type [32]. Consistent with these observations, we found all of the cannabis photosynthetic tissues to have similar expression profiles (Figure 3a).

Nonetheless, flowers show a pattern of gene expression consistent with the biosynthesis of cannabinoids and terpenoids in these organs. Cannabinoids are prenylated polyketides that are synthesized from the short-chain fatty acid hexanoate and geranyl diphosphate (GPP) [23,33]. The latter precursor, which is the substrate for an aromatic prenyltransferase enzyme, is derived from the 2-*C*-methyl-D-erythritol 4-phosphate (MEP) pathway [34-36] (see Figure 3b for details). We found that the genes encoding cannabinoid pathway enzymes and also most of those encoding proteins (e.g. hexanoate, MEP and GPP) involved in putative precursor pathways were most highly expressed in the three stages of flower development (pre-flowers, and flowers in early and mid-stage of development) (Figure 3c). This finding is consistent with cannabinoids being synthesized in glandular trichomes, the highest density of which is found on female flowers [37]. The production of THCA in marijuana strains (such as PK) and CBDA in hemp, is due to the presence or absence of THCAS and CBDA synthase (CBDAS) in these two chemotypes. Indeed, THCAS is highly expressed in PK flowers of all stages, whereas CBDAS is absent (Figure 3c).

It is worth noting that of the 19 'pathway genes' we analyzed, 18 were complete in the transcriptome assembly, underscoring its quality. The transcript of the *MDS *gene (which encodes a protein involved in the MEP pathway) was assembled in two fragments with a blunt overlap of 48 nt, narrowly missing the merging threshold of 50 nt. This sequence was resolved by merging the fragments manually. All 'pathway genes' are fully represented in the draft genome and an overview of their genomic locations is provided on the Cannabis Genome Browser website [30].

### Comparison of the expression of cannabinoid pathway genes between marijuana (PK) and hemp ('Finola')

**Figure 4:**
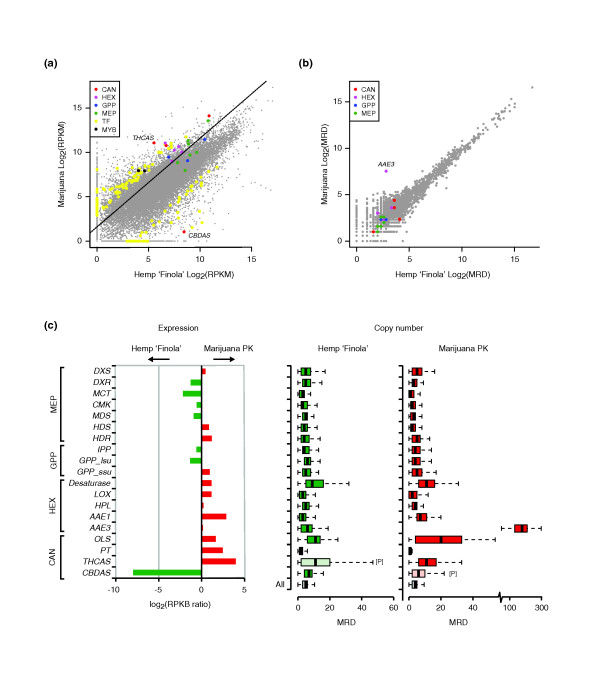
**Comparison of gene expression in female cannabis flowers, and gene copy number, between marijuana (PK) and hemp ('Finola')**. (**a**) A scatter plot of RNA-Seq read counts for all representative transcripts in marijuana and hemp, expressed as log2 RPKM. Specific subsets of transcripts are shown in color, as indicated in the key. The dashed line represents the relative enrichment of trichomes in the marijuana strain, inferred from the ratio in expression of trichome-specific genes, as defined in the text. Gene symbols/abbreviations: CAN - known and putative cannabinoid pathway genes; HEX - putative hexanoate pathway genes; GPP - GPP pathway genes; MEP - MEP pathway genes; TF - putative transcription factors according to PFAM, with at least a 4-fold change in expression in PK relative to 'Finola'; MYB - Myb-domain transcription factors previously suggested as trichome regulators. (**b**) A scatter plot of the log2 median read depth (MRD) of genomic DNA-Seq reads that aligned uniquely to the PK transcriptome. Genomic reads were trimmed to a length of 32 bases prior to alignment with Bowtie, to allow for mapping close to exon junctions. The lack of outliers in the scatter plot indicates that there have been relatively few changes in gene copy number between marijuana and hemp. (**c**) The relative RNA-Seq expression of individual genes in the cannabinoid pathway and precursor pathways (is shown on the left), adjusted for enrichment of trichome-specific genes (i.e. relative to the dashed line in panel a). The median genomic DNA read depth for the same genes is shown on the right. The box plots reflect the variation in the depth of coverage of uniquely aligned genomic DNA reads across each transcript, with the median coverage distribution across all transcripts shown as reference (All). Reads that are likely derived from pseudogenes are marked by the symbol [P]. While there is increased expression of most cannabinoid genes in the HEX and CAN pathways (left) in PK, this does not appear to be due to an increased representation of these genes in the PK genome relative to the 'Finola' genome (right).

Although there are differences in the morphology of marijuana and hemp strains, the THC content of PK and other strains selected and bred for use as marijuana is remarkably high. We investigated whether the high THC production in PK was associated with increased gene expression levels of cannabinoid pathway enzymes, relative to those in hemp. We performed RNA-Seq analysis on Finola flowers at the mid-stage of development, generating a total of 18.2 M reads. 'Finola' is a short, dioecious, autoflowering cultivar developed in Finland for oil seed production. It was created by crossing early maturing hemp varieties from the Vavilov Research Institute (St. Petersburg, Russia), 'Finola' might be derived from a "*ruderalis*" genetic background [38]. It contains moderate amounts of CBDA in female flowers but very low amounts (< 0.3% by dry weight) of THCA. Figure 4a shows that the overall mid-flower transcript profiles, expressed as RPKM (reads per kb per million reads), are similar between PK and 'Finola'; however, the entire cannabinoid pathway is expressed at higher levels in PK than in 'Finola', with later steps increased as much as 15-fold (Figure 4a).

The difference in gene expression is not due to morphological variability between the strains, such as in the size or proportion of trichomes. We examined the global expression levels of trichome genes to account for possible differences in trichome density between PK and 'Finola' flowers, by analyzing an RNA-Seq dataset obtained for 'Finola' glandular trichomes (from a separate study, data not shown). From a set of the1000 most abundant transcripts, we selected 100 that had the greatest difference in expression between the mid-flower stage and the maximum expression level found in PK root, shoot or stem in the current study. This subset of genes should be highly enriched for genes predominantly expressed in glandular trichomes (and indeed contained all the cannabinoid and hexanoate 'pathway genes' expressed in 'Finola'). The median difference in expression level after excluding the cannabinoid genes is shown as a dotted line in Figure 4a, and was used to adjust the expression differences shown in Figure 4c. Even after accounting for global trichome differences, cannabinoid pathway enzymes remain among several hundred obvious outliers. Outliers also include several dozen transcription factors, including two Myb-domain proteins that have been previously suggested to play a role in regulating processes in cannabis trichomes [23] (Figure 4a). These data suggest that the increased production of cannabinoids in PK may be due in part to an increase in expression of the biosynthetic genes.

### Resequencing of the C. sativa 'Finola' genome reveals copy number changes in a PK cannabinoid pathway related enzyme

To begin our search for the underlying causes of the differences between marijuana and hemp, we sequenced the genome of 'Finola' (e.g. Illumina 100 nt paired-end reads, 200-500 bp inserts, at approximately 50× coverage of the estimated 820 Mb genome). Plant genomes often contain many duplicated genes, and gene amplification represents a well-documented mechanism for increasing expression levels [39]. Therefore, we first asked whether there were apparent differences in copy number for the enzyme-encoding gene set, using the median read depth (MRD) of genomic DNA-Seq reads that could be uniquely mapped to transcripts as a proxy. Figure 4b illustrates that, overall, there appear to be relatively few differences in gene MRD between PK and 'Finola'. The exception to this is the much expanded coverage for *AAE3*, a gene encoding an enzyme of unknown function in PK. *AAE3 *is similar to an *Arabidopsis AAE *[TAIR:At4g05160] that has been shown to activate medium- and long-chain fatty acids including hexanoate [40]. Although *AAE1 *is a more likely candidate for the hexanoyl-CoA synthetase involved in cannabinoid biosynthesis (JMS and JEP, unpublished results), owing to its high expression in flower tissues and increased transcript abundance in PK (Figure 3b, 4), *AAE3 *might play an, as yet, unknown role in cannabinoid biosynthesis. Because we could detect both multi- and single-exon copies of AAE3, we believe that the large expansion of *AAE3 *has occurred through the insertion of processed pseudogenes in the PK genome. In addition, the read depth analysis uncovered reads corresponding to *CBDAS *in PK and *THCAS *in 'Finola'. However, on the basis of our inability to assemble these into functional protein-coding genes, we conclude that the *THCAS *reads in 'Finola' and *CBDAS *reads in PK are likely to be caused by the presence of pseudogenic copies, as we discuss below. Therefore, it appears that the differences in expression of cannabinoid pathway enzymes between marijuana and hemp are due to subtle genetic differences that cause changes in gene expression, either directly or indirectly.

The PK genome contains two copies of two genes involved in cannabinoid biosynthesis. Copies of *AAE1*, which encodes a protein likely to synthesize the hexanoyl-CoA precursor for cannabinoid biosynthesis, are found on scaffold1750 [genbank:JH227821] and scaffold29030 [genbank:JH245535]. *OLS*, which encodes the putative cannabinoid pathway polyketide synthase [18], was found to be duplicated at scaffold15717 [genbank:JH226441] and scaffold16618 [genbank:JH237993].

### Analysis of single nucleotide variants (SNVs) in cannabis

To further examine the genetic variation in *C. sativa*, we estimated the frequency of SNVs in four taxa. In addition to PK and 'Finola', our analysis included the Illumina reads we generated from the hemp cultivar 'USO-31', as well as the reads from the marijuana strain Chemdawg, which were released by Medical Genomics, LLC [41] while this manuscript was in preparation. 'USO-31' is a tall, monoecious fibre hemp cultivar developed in the former Soviet Union that contains very low or undetectable levels of cannabinoids [42]. Our resequencing of 'USO-31' was similar to that of 'Finola' (Illumina 100 nt paired-end reads, 200 and 500 bp inserts, at approximately 16× coverage of the estimated 820 Mb genome). We aligned individual Illumina reads to the PK reference genome, and identified variant bases that qualify as SNVs (see the Methods section for further details). We also quantified the degree of heterozygosity within single genomes. Overall, PK, Chemdawg, 'Finola' and 'USO-31' have comparable rates of heterozygosity (0.20%, 0.26%, 0.25%, and 0.18%, respectively). The lower rate of heterozygosity in 'USO-31', which is monoecious, is presumably due to self-pollination.

**Figure 5:**
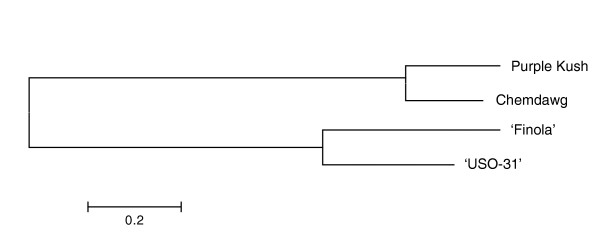
**Neighbour-joining tree for two hemp cultivars and two marijuana strains**. The tree was plotted in MEGA5 [71] using the maximum composite likelihood of SNV nucleotide substitution rates, calculated based on the concatenated SNV sequences in each variety, as a distance metric. The topology of the tree reveals a distinct separation between the hemp and marijuana strains.

The rate of occurrence of SNVs between any two strains ranged from 0.38% (PK versus Chemdawg) to 0.64% (Chemdawg versus 'Finola'). A neighbor-joining tree constructed using the concatenated polymorphic sequences from each of the strains is shown in Figure 5, and supports the expected pedigree of the two hemp cultivars, which are likely to have been bred using germplasm from North Central Asia. Although the ancestry of PK and Chemdawg is unclear, their position on the tree supports the notion that marijuana forms of cannabis are more related to each other than to the hemp forms, and that these two marijuana strains share a common heritage.

### Genomic analysis of cannabinoid chemotypes

The molecular basis for THCA (marijuana) and CBDA (hemp) chemotypes is unclear. De Meijer *et al *[43] crossed CBDA- and THCA-dominant plants to produce F1 progeny that are intermediate in their ratio of THCA:CBDA; selfing gave F2 progeny that segregated 1:2:1 for THCA-dominant:codominant mixed THCA/CBDA:CBDA-dominant chemotypes. These data suggested two explanations: a single cannabinoid synthase locus (*B*) exists with different alleles of this gene encoding THCAS or CBDAS; or THCAS and CBDAS are encoded by two tightly linked yet genetically separate loci. In the latter scenario, differences in transcript abundance and/or enzyme efficiencies might account for the observed chemotypic ratios. Indeed, given that both of these enzymes compete for CBGA, reductions in one activity might lead to a proportional increase in the production of the other cannabinoid. Our draft sequence of the THCA-dominant PK genome enables some preliminary insights into possible mechanisms of the inheritance of cannabinoid profiles. Using the published *THCAS *sequence [genbank:AB057805] [16] to query the PK genome, a single scaffold of 12.6 kb (scaffold19603, [genbank:JH239911]) was identified that contained the *THCAS *gene as a single 1638 bp exon with 99% nucleotide identity to the published *THCAS *sequence. Querying the PK transcriptome returned the same *THCAS *transcript (PK29242.1, [genbank:JP450547]) that was found to be expressed at high abundance in female flowers (Figure 3c). A *THCAS-like *pseudogene (scaffold1330 [genbank:JH227480], 91% nucleotide identity to *THCAS*) was also identified. We used the *CBDAS *sequence [genbank:AB292682] [17] to query the PK genome and identified as many as three scaffolds that contain *CBDAS *pseudogenes (scaffold39155 [genbank:AGQN01159678], 95% nucleotide identity to *CBDAS*; scaffold6274 [genbank:JH231038] + scaffold74778 [genbank:JH266266] combined, 94% identity; and scaffold99205 [genbank:AGQN01254730], 94% identity), all of which contained premature stop codons and frameshift mutations. The presence of these pseudogenes in the PK genome accounts for the identification of *CBDAS *genomic sequences in PK (Figure 4c). A 347-bp transcript fragment (PK08888.1, [genbank:JP462955]) with 100% nucleotide identity to *CBDAS *could be identified in the PK transcriptome, likely due to the nonsense-mediated decay of transcripts derived from *CBDAS *pseudogenes. Given that multiple independent loci were identified with high sequence similarity to either *THCAS *or *CBDAS *in a THCA-dominant marijuana strain, the two-loci model for the genetic control of THCA:CBDA ratios might be correct. A possible explanation is that during the development of high-THC marijuana strains such as PK, underground breeders selected for non-functional *CBDAS *that would effectively eliminate substrate competition for CBGA and thus increase THCA production. Alternatively, the *CBDAS *pseudogene in the PK genome might occur in all cannabis strains. If this is true, the single-locus model might still be correct, and we did not find a CBDAS-encoding allele at this locus because PK is homozygous for *THCAS*.

### Analysis of PK transcriptome for cannabichromenic acid synthase (CBCAS) candidates

To illustrate the potential value of the cannabis genome and transcriptome to elucidate cannabinoid biosynthesis, we searched for genes encoding enzymes that might catalyze the formation of cannabichromenic acid (CBCA), which is present in most cannabis plants as a minor constituent and in certain strains as the dominant cannabinoid [44]. Although a protein that synthesizes CBCA has been purified from cannabis, the gene that encodes the CBCA synthase (CBCAS) has not been identified [45]. We hypothesized that CBCAS is an oxidocyclase enzyme related to THCAS and CBDAS, therefore, we queried the PK transcriptome using *THCAS *and *CBDAS *sequences. In total, 23 candidates were identified that had greater than 65% nucleotide identity with these sequences. These include four genes that we designated *THCAS-like1 *to *THCAS-like4*, which encode proteins that are 89%, 64%, 68%, and 59% identical to THCAS at the amino acid level, respectively. We also identified transcripts corresponding to *CBDAS2 *and *CBDAS3*, which are closely related to *CBDAS *but do not encode enzymes with CBDAS activity [17]. The remaining 18 transcripts encode proteins that are similar to reticuline oxidase, an oxidoreductase that functions in alkaloid biosynthesis [46]. Biochemical analysis of CBCAS candidates is currently underway.

## Discussion

We anticipate that the cannabis genome and transcriptome sequences will be invaluable for understanding the unique biological properties and considerable phenotypic variation in the genus *Cannabis*. These genomic resources are applicable to the molecular analysis of both marijuana and hemp, as we sequenced a marijuana strain (PK) and two hemp cultivars ('Finola' and 'USO-31') grown in Canada and elsewhere. The high repeat content of plant genomes, coupled with the relatively high level of sequence variation in cannabis [47-49], complicates the assembly of the full genome into the anticipated nine autosomes and two sex chromosomes. We will continue to explore approaches that might facilitate assembly of the full genome sequence, including anchoring the genome using molecular markers or FISH (fluorescence *in situ *hybridization) [50]. A more complete assembly might provide the sequences of the × and Y chromosomes and help shed light on the mechanism of sex determination in cannabis. Nonetheless, our current assembly appears to encompass the vast majority of the non-repetitive genome and the individual genes.

Mechoulam [13] characterized the plant-derived cannabinoids as a 'neglected pharmacological treasure trove' and others have noted the potentially useful biologically activities yet to be identified for this group of plant natural products [15]. Medical marijuana strains reportedly have different therapeutic effects based on levels of THC, THC:CBD ratios, the presence of minor cannabinoids and the contribution of other metabolites such as terpenoids [51]. The sequences of the cannabis genome and transcriptome will provide opportunities for identifying the pathways and remaining enzymes leading to the major and minor cannabinoids. Such knowledge will facilitate breeding of cannabis for medical and pharmaceutical applications. For example, analysis of the PK transcriptome has enabled us to identify several candidate genes that might encode CBCAS, which forms a cannabinoid with interesting biological activities [14,52,53]. Despite the low levels of THC in modern hemp strains, the cannabinoid content of hemp remains a significant impediment to wider cultivation because of regulations that require germplasm to be carefully controlled and for crops to be tested to ensure they contain less than 0.3% THC. The genome sequence will aid the development of hemp cultivars that are devoid of cannabinoids through marker-assisted selection and other breeding techniques.

The differences in the expression of cannabinoid pathway enzymes in PK and 'Finola' are also of interest, and could be due to either cis- or trans-regulatory alterations. The up-regulation of the cannabinoid pathway in PK appears to be a consequence of the longstanding breeding effort to create marijuana strains with enhanced psychoactivity through increased THC levels. Plant domestication is often accompanied by a reduction of secondary metabolic pathways, many of which produce toxic or unpalatable compounds that have defensive functions [54,55]. The opposite appears to be the case in marijuana strains of cannabis, where there has been positive selection for THC production. This is primarily due to two molecular events: the selection for *THCAS *over *CBDAS *and the up-regulation of the cannabinoid pathway and pathways supplying metabolic precursors. Our analysis indicates that amplification of cannabinoid pathway genes does not appear to play a causative role in this increased expression. Most of the key domestication genes in crop plants have been shown to encode transcription factors [56]. It seems likely that one of the processes causing the emergence of high-THC marijuana strains is also due to transcriptional alterations in cannabinoid pathway regulation. Indeed, we find evidence for the increased expression of a multitude of transcription factors in PK compared with those in 'Finola' (Figure 4a).

The underlying mechanisms for this transcriptional control could probably be dissected using existing techniques, were there not severe legal restrictions in most jurisdictions on growing cannabis, even for research purposes. Although this difficulty is somewhat unique to cannabis, more generally it is becoming common to obtain genome sequences and transcriptome data for organisms that are not experimentally tractable. We propose that *in silico *analyses, for example, modeling of regulatory networks, can provide a way to explore the function and evolution of such genomes. On the basis of close homology to *Arabidopsis *transcription factors, it is possible to infer the sequence specificities of many cannabis transcription factors (HvB and M Weirauch, unpublished results). This modeling of cannabis transcriptional networks is already feasible.

Finally, the genome sequence will enable investigation of the evolutionary history, and the molecular impact of domestication and breeding on *C. sativa*. The taxonomic treatment of the genus *Cannabis *has been controversial. It might be feasible to use sequence-based genotyping to trace the relationships in cannabis taxa, including wild germplasm, landraces, cultivars and strains, as has recently been demonstrated in grape [57,58]. Our SNV analysis has already allowed for the separation of two hemp cultivars from two marijuana strains, suggesting additional analysis of diverse cannabis germplasm is warranted. Outstanding areas that might be addressed by further genomic investigation include whether the genus is composed of one or several species, the existence of 'sativa' and 'indica' gene pools, the relative contributions that wild ancestors have made to modern hemp and marijuana germplasm, and the process by which cannabis was first domesticated by humans.

## Conclusions

*C. sativa *has been cultivated throughout human history as a source of fibre, oil, food, drugs and medicine. Here, we have presented a draft genome and transcriptome of *C. sativa*, and compared the genomes and flower transcriptomes of high- and low-THCA producing strains (PK (high), 'Finola' (low) and 'USO-31' (low to absent)). *THCAS*, the gene encoding the oxidocyclase enzyme that forms the THC precursor THCA, is found in the genome and transcriptome of PK, whereas *CBDAS *dominates in the 'Finola' hemp cultivar. Moreover, we find that most of the cannabinoid biosynthetic pathway enzymes are highly expressed in flower tissues containing glandular trichomes, and that the expression of the cannabinoid biosynthetic enzymes is elevated in the high-THCA PK strain, even relative to other genes expressed specifically in glandular trichomes. Although some of the genes encoding pathway enzymes are present in multiple copies, amplifications do not appear to account for the increased expression. The *C. sativa *genome sequence will greatly facilitate exploration of the molecular biology and evolutionary history of this culturally significant and exceptionally useful plant.

## Materials and methods

### Plant material

Frozen samples of *C. sativa *PK from clonally propagated female plants were obtained from an authorized medical marijuana grower in Vancouver, BC, Canada. Plants of the hemp cultivars *C. sativa *'Finola' (originally called 'FIN-314') and *C. sativa *'USO-31' were grown from seed in controlled environment chambers at the NRC Plant Biotechnology Institute, Saskatoon, SK, Canada.

### Nucleic acid isolation

Genomic DNA was extracted from nuclei isolated from approximately 30 g of young leaves from PK using the method described in [59] with modifications from [60]. DNA was isolated from a single 'Finola' plant and a single 'USO-31' plant using the same method. For RNA-Seq analysis, total RNA was isolated from PK roots, stems, shoots (shoot tips with young leaves and apical meristems), pre-flowers (shoot tips with flower primordia but no visible stigmas), and early-stage flowers (flowers with visible stigmas) and mid-stage flowers (flowers with visible, non-withered stigmas and conspicuous trichomes). A CTAB-based method [61] followed by clean-up with an RNeasy Plant Mini Kit (Qiagen, Venlo, Netherlands) was used. Genomic DNA was removed by on-column digest with DNase I (Qiagen). Total RNA was isolated from 'Finola' mid-stage female flowers using the same method.

### Illumina paired-end library construction and sequencing

Paired-end genomic DNA libraries were constructed using reagents from the NEBNext DNA Sample Prep Reagent Set 1 (New England Biolabs, Ipswich, MA, USA) or the Paired-End DNA Sample Prep Kit (Illumina, San Diego, CA, USA). Genomic DNA (5-10 μg) was sheared using the Bioruptor Standard sonication device (Diagenode, Liège - Belgium) for 20 min on low power using 30 s 'ON' and 'OFF' cycles. Fragmented DNA was purified using a PCR purification kit (Qiagen), and was subjected to an end-repair reaction for 30 minutes at 20°C containing 1× end-repair buffer, 0.4 mM dNTPs, 5 μl T4 DNA Polymerase, 1 μl Klenow Large Fragment, and 5 μl T4 PNK in a final reaction volume of 100 μl. The reaction was then purified using the Qiagen PCR purification kit and the 3'-ends of the DNA were adenylated for 30 min at 37°C in a reaction containing 1× Klenow buffer, 0.2 mM dATP, and 3 μl Klenow (Exo-) in a final volume of 50 μl. Adenylated DNA was again purified using the Qiagen MinElute PCR purification kit. Adapters were ligated to the purified DNA for 30 min at 20°C in a reaction containing 1× T4 DNA ligase buffer with ATP, 0.3 μM Adapter Oligo Mix, and 5 μl T4 DNA Ligase in a final volume of 50 μl. Ligation reactions were immediately analyzed on a 1% agarose gel and bands of the desired size were excised and purified using the QIAQuick Gel Extraction Kit (Qiagen). 1-2 μl of the purified DNA was used in a PCR reaction containing 1× Phusion buffer, 0.2 mM dNTPs, 0.5 μM each of PCR primers PE 1.0 and 2.0 and 2 U Phusion DNA Polymerase in a final reaction of 50 μl. Thermal cycler conditions were as follows: 98°C for 30 s, 10 cycles of 98°C for 15 s, 65°C for 30 s, and 72°C for 30 s, followed by 72°C for 5 min. PCR reactions were run on a 1% agarose gel and fragments of the desired size were excised and purified using the QIAQuick Gel Extraction Kit. Products were quantified using the Bioanalyzer 2100 (Agilent, Santa Clara, CA, USA) and the KAPA Library Quantification Kit for Illumina (KAPA Biosystems, Woburn, MA, USA), and sequenced as 2 × 100 nt paired-end reads on the Genome Analyzer IIx or Hi-Seq instruments (Illumina).

### Illumina mate-pair library construction and sequencing

The 2-kb and 5-kb mate pair libraries were prepared using the Mate Pair Library Preparation Kit v2 (Illumina). 10 μg genomic DNA was fragmented using the S2 Adaptive Acoustic Device (Covaris, Woboum, MA, USA) following the manufacturer's recommendations. The fragmented DNA was subjected to an end-repair reaction for 30 min at 20°C containing 1× end-repair buffer, 1.5 μl mM dNTPs, 2.5 μl biotinylated dNTPs, 5 μl T4 DNA Polymerase, 1 μl Klenow Large Fragment, and 5 μl T4 PNK in a final reaction volume of 100 μl. The DNA was then run on a 0.8% agarose gel and bands of the desired size were excised and purified using the QIAQuick Gel Extraction Kit (Qiagen). 600 ng of purified DNA was circularized overnight at 30°C in a reaction containing 1× Circularization Buffer and 13.4 μl Circularization Ligase in a total volume of 300 μl. The next day, 3 μl DNA exonuclease was added to the reaction and incubated for 20 min at 37°C. The circularized DNA was fragmented using the Bioruptor sonication device as described above. The fragmented DNA was then applied to DynaI magnetic M-280 beads (Invitrogen, Carlsbad, CA, USA) and washed as recommended by the manufacturer to enrich for biotinylated DNA fragments. The fraction of DNA bound to the beads was subjected to end-repair, adenylation, and adapter ligation as described above, except that each step was followed by a bead wash instead of column purification. The beads were resuspended in 50 μl PCR mix (1× Phusion buffer, 0.2 mM dNTPs, 0.5 μM each of PCR primers PE 1.0 and 2.0 and 2 U Phusion DNA Polymerase). Thermal cycler conditions were as described above, except that 18 cycles were used. Size selection, gel purification, and quantification of libraries were as described above. Mated libraries were sequenced as 2 × 42 nt reads on an Illumina HiSeq instrument.

### cDNA library construction and sequencing

Normalized, full length-enriched cDNA was generated from total RNA pooled from PK tissues by Bio S&T (Montreal, QC, Canada) and the resulting double-stranded cDNA was fragmented. Libraries were generated as described for the paired-end genomic DNA libraries. mRNA-Seq libraries from individual plant tissues were prepared by the Virginia Bioinformatics Institute (Blacksburg, VA, USA). All cDNA libraries were sequenced as single-end 100 nt reads (individual tissues) or paired-end 100 nt reads (normalized sample) on Genome Analyzer IIx or Hi-Seq instruments (Illumina).

### 454 library construction and sequencing

To construct the paired-end libraries for 454 sequencing, we followed the method described in the GS FLX Titanium 20 kb and 8 kb Span Paired End Library Preparation Method Manual from Roche (April 2009 version; Roche, Basel, Switzerland) with the following modifications. In Manual section 3.1, high-quality genomic DNA (45 μg) was fragmented using a Hydroshear (Digilab, Holliston, MA, USA) with the large assembly and set to speed code 18 for 20 cycles. In section 3.3, the fragmented DNA was separated on a single lane of a 0.5% agarose gel (Megabase, from Bio-Rad, Hercules, California, USA) in 1× TAE for 16 h at 14°C using a FIGE Mapper Electrophoresis System (Bio-Rad). The switch time ramp was set at 0.1-0.8 s with a linear shape and forward and reverse voltages were 180 V and 120 V, respectively. In section 3.4, four slices were cut from the one lane (13 kb, 20 kb, 30 kb and 40 kb). Libraries were multiplied at the DNA circularization (section 3.6) and library amplification (section 3.10) steps. For circularization of the 40 kb libraries, 600 ng were used instead of 300 ng (section 3.6.2). During the circularization incubation program (section 3.6.5), the 40-kb libraries were held at 37°C for 60 min instead of 45 min. To make the DNA beads, we followed the method described in the emPCR Method Manual - Lib-L LV GS FLX Titanium Series (Roche, October 2009 (Rev. Jan 2010)). For emPCR of paired-end libraries, the Live Amplification Mix (section 3.1.4) was modified with addition of smaller volume of amplification primer and the heat denaturation (section 3.2.6) was omitted. To sequence, we followed the method described in the Sequencing Method Manual GS FLX Titanium Series (Roche, October 2009 (Rev. Jan 2010)) with software v2.5.3.

### De novo genome assembly

All Illumina reads were filtered on quality; allowing for no more than 10 bp with a Phred quality score below 30; discarding the rest of the sequence and keeping only pairs where both reads were larger than 55 bp. Next we used cutadapt [62] to remove any reads that were contaminated with an Illumina adapter. Bacterial reads were removed by aligning each Illumina library to all sequenced bacterial genomes using Bowtie v0.12.7 [63]. For the Illumina mate pair data, we estimated the proportion of unmated reads to range from 4.6 to 7.8%, based on a comparison of the number of reads that mapped to contigs > 10 kb in a reverse-forward orientation (mated) to the number of reads mapping in a forward-reverse orientation (unmated). To remove unmated reads, we used Bowtie to map the mate pair libraries to the Illumina paired-end reads with inserts ranging between 200 and 660 bp, and discarded those that were fully contained within a single short-insert read pair. This procedure reduced the proportion of unmated reads to < 0.2%. Finally we discarded duplicated mate pairs with identical sequence in the first 30 bp of both reads, which accounted for < 3% of the data. For 454 data, we used the CABOG [64] tool sffToFrg to identify mated reads and remove duplicate mate pairs. The proportion of duplicates in the 454 libraries ranged from 3.1% to 12.2%. The remaining read sequence data were converted to fastq format, trimmed to a length of 65 bases and used in combination with the Illumina reads in the assembly.

The genome was assembled with SOAPdenovo v1.0.5 using a kmer parameter of 39, which was selected after testing a range of kmers settings between 31 and 41, and a merge level of two. The mate pair libraries were only incorporated during the scaffolding phase, using a cut-off of three or four mapped pairs to identify reliable links between contigs for the 454 and Illumina mate libraries, respectively. The SOAPdenovo 'GapCloser' tool was used with default settings after scaffolding, closing 166 Mb of gaps. Following assembly, we identified near-identical scaffolds that shared ≥ 98% identity across ≥ 95% of the length of the smallest scaffold. We assessed that these occurrences represented instances where heterozygosity resulted in distinct assemblies of each strand and therefore selected the largest scaffold as the representative genome sequence. Finally, we removed potential bacterial contigs by aligning the draft assembly to all available fully sequenced microbial genomes obtained from NCBI in April 2011, and removing scaffolds with significant blat or blastx hits (score > 150) and a median read coverage more than 2 SDs outside the range observed for validated *Cannabis sativa *scaffolds with high sequence similarity to other plant genomes.

### De novo transcriptome assembly

Each tissue and normalized RNA-Seq library, as well as a combination of all libraries, was assembled with ABySS v1.2.6 and/or Inchworm v03132011. For ABySS we used the following parameters: k - 49, e - 5, n - 5. For Inchworm we set the k-mer size to 31. These parameters were optimized for scaffold N50 and total base coverage, after running each assembler across a range of parameters.

Following assembly, we used three different approaches to QC and identify the coding strand of each transcript. First, each transcript was compared with the predicted ORF translations for three fully sequenced plant genomes (*Arabidopsis *release TAIR10, Maize release ZmB73_5b, and Rice release 6.1) using blastx [65]. We selected for PK transcripts matching at least one ORF translation with an e-value below 10^-6 ^and used the alignment strand information to orient the transcript. In case multiple blastx hits were found on conflicting strands, the transcript was dropped from the assembly. Second, we identified transcripts that had an open reading frame ≥ 240 nt spanning ≥ 70% of the length of the transcript on one strand, with the additional requirement that there was a ≥ two-fold difference in size compared with any ORF found on the opposite strand. Transcripts meeting these criteria were oriented according to the strand containing the largest ORF. Third, we used blat [66] to align the transcriptome assemblies to the genome assembly to identify spliced transcripts. We selected alignments where a transcript matched a genomic contig in consecutive blocks with ≥ 95% overall sequence identity and a minimum block (i.e. exon) size of 30 nt. The boundaries of aligned blocks were considered candidate splice sites and further examined for the presence of the canonical splice junction donor/acceptor sequences (GT/AG for the coding strand and CT/AC for the template strand, respectively) in the genomic scaffolds sequence directly adjacent to the aligned blocks. We selected transcripts with at least one candidate splice site matching the canonical junction sequences, while discarding those that had an equal or greater number of candidate splice sites that did not match the canonical sequences. The resulting set of transcripts was then oriented according to the directionality of the splice junction sequences.

The transcripts that met the criteria for at least one of the three methods outlined above, and that had no conflicting orientation information between these methods, were selected for each assembly (see Table 2 for a summary of each assembly at this stage). Overall, 64% of all transcripts had their orientations derived from two or more methods. We then combined the filtered and oriented transcripts from each assembly together and used cd-hit-est [67] to merge transcripts between assemblies when they shared ≥ 97% identity across ≥ 95% of the length of the smallest transcript, keeping the largest transcript in each cluster. Given that we frequently observed chimeric transcripts in the Inchworm assembly, we only included Inchworm transcripts that were covered for more than 70% of their length by a single blast hit during the merging stage. Finally, we used blat to identify blunt overlaps of at least 50 nt between transcript fragments and used cap3 [68] to join these fragments together. From this assembly we selected an additional set of representative transcripts by first clustering overlapping isoforms when they shared ≥ 95% similarity across ≥ 100 nucleotides, and then choosing the transcript with the largest ORF (Table 2). Finally, remaining traces of vector or adapter contamination were removed by screening against the UniVec database.

### Variant analysis

A subset of the QC filtered Illumina genomic DNA libraries was selected to obtain an estimated 30× coverage of sequence data for PK, Chemdawg and 'Finola', as well as 16× coverage of 'USO-31'. Each dataset was aligned to the PK genome assembly using Bowtie v0.12.7 [63] and variants were called across the four sets using the multi-sample mpileup option in SAMtools (v0.1.17) [69]. We selected for SNVs with a quality score ≥ 30, corresponding to a ≤ 10^-3 ^likelihood of an incorrect call. In addition, we restricted our analysis to regions uniquely covered by at least five reads in each cultivar and no more than 150 reads across all samples combined (a total of 159 Mb of the PK reference genome), to restrict our analysis to regions where we had data for all cultivars, and to limit spurious calls in repetitive regions of the genome.

## Accession numbers

The PK Whole Genome Shotgun project has been deposited at DDBJ/EMBL/GenBank under the accession [genbank:AGQN00000000]. The version described in this paper is the first version, [genbank:AGQN01000000], and corresponds to the canSat3 assembly in the Cannabis Genome Browser. Assembled transcripts ≥ 200 nt have been deposited in the NCBI Transcriptome Shotgun Assembly (TSA) sequence database with accession numbers between [genbank:JP449145] - [genbank:JP482359]. Raw sequence read data have been deposited in the NCBI Sequence Read Archive with the following study identifiers: PK genomic DNA - [SRA:SRP008673]; PK RNA-Seq - [SRA:SRP008726]; 'Finola' genomic DNA - [SRA:SRP008728]; 'Finola' RNA-Seq - [SRA:SRP008729]; 'USO-31' genomic DNA - [SRA:SRP008730].

## Abbreviations

AAE: acyl-activating enzyme; bp: base pair; BP: before present; CBC: cannabichromene; CBCA: cannabichromenic acid; CBCAS: cannabichromenic acid synthase; CBD: cannabidiol; CBDA: cannabidiolic acid; CBDAS: cannabidiolic acid synthase; CBGA: cannabigerolic acid; CMK: 4-diphosphocytidyl-2-C-methyl-D-erythritol kinase; CTAB: cetyl trimethylammonium bromide; DXR: 1-deoxy-D-xylulose 5-phosphate reductoisomerase; DXS: 1-deoxyxylulose-5-phosphate synthase; EST: expressed sequence tag; FIGE: Field inversion gel electrophoresis; FISH: fluorescence in situ hybridization; Gb: giga base pair; GPP: geranyl diphosphate; GPP synthase lsu: GPP synthase large subunit; GPP synthase ssu: GPP synthase small subunit; HDR: 4-hydroxy-3-methylbut-2-enyl diphosphate reductase; HDS: 4-hydroxy-3-methylbut-2-en-1-yl diphosphate synthase; HPL: hydroperoxide lyase; kb: kilo base pair; LOX: lipoxygenase; Mb: mega base pair; MCT: 4-diphosphocytidyl-methylerythritol 2-phosphate synthase; MDS: 2-C-methyl-D-erythritol 2:4-cyclodiphosphate synthase; MEP: 2-*C*-methyl-D-erythritol 4-phosphate; ORF: open reading frame; OLS: olivetol synthase; PK: Purple Kush; PT: prenyltransferase; QC: quality control; RPKM: reads per kb per million reads; SNV: single nucleotide variants; THC: Δ^9^-tetrahydrocannabinol; THCA: Δ^9^-tetrahydrocannabinolic acid; THCAS: Δ^9^-tetrahydrocannabinolic acid synthase; THCV: Δ^9^-tetrahydrocannabivarin.

## Competing interests

The authors declare that they have no competing interests.

## Authors' contributions

TRH and JEP conceived of the project. HvB performed the genome and transcriptome assembly and generated the figures. JMS and JEP extracted the nucleic acids. AGC prepared the Illumina sequencing libraries. CMT prepared and sequenced the 454 libraries under the direction of AGS. TRH, JEP, HvB and JMS wrote the manuscript. All authors have read and approved the manuscript for publication.

## References

[B1] Schultes RE Klein WM Plowman T Lockwood TE Cannabis: an example of taxonomic neglect. Bot Mus Leafl Harvard Univ 1974 23 337 367

[B2] Li HL An archaeological and historical account of cannabis in China. Econ Bot 1973 28 437 444 10.1007/BF02862859

[B3] Russo EB Jiang H-E Li X Sutton A Carboni A Bianco F del Mandolino G Potter DJ Zhao Y-X Bera S Zhang Y-B Lü E-G Ferguson DK Hueber F Zhao L-C Liu C-J Wang Y-F Li C-S Phytochemical and genetic analyses of ancient cannabis from Central Asia. J Exp Bot 2008 59 4171 4182 10.1093/jxb/ern260 19036842 PMC2639026

[B4] Zias J Stark H Sellgman J Levy R Werker E Breuer A Mechoulam R Early medical use of cannabis. Nature 1993 363 215 8387642 10.1038/363215a0

[B5] UNODC World Drug Report 2011 United Nations Publication, Sales No. E.11.XI.10

[B6] Ware MA Wang T Shapiro S Robinson A Ducruet T Huynh T Gamsa A Bennett GJ Collet J-P Smoked cannabis for chronic neuropathic pain: a randomized controlled trial. CMAJ 2010 182 E694 701 10.1503/cmaj.091414 20805210 PMC2950205

[B7] Lakhan SE Rowland M Whole plant cannabis extracts in the treatment of spasticity in multiple sclerosis: a systematic review. BMC Neurol 2009 9 59 10.1186/1471-2377-9-59 19961570 PMC2793241

[B8] Hillig K Genetic evidence for speciation in *Cannabis *(Cannabaceae). Genet Resourc Crop Evol 2005 52 161 180 10.1007/s10722-003-4452-y

[B9] Elsohly MA Slade D Chemical constituents of marijuana: The complex mixture of natural cannabinoids. Life Sci 2005 78 539 548 10.1016/j.lfs.2005.09.011 16199061

[B10] Mehmedic Z Chandra S Slade D Denham H Foster S Patel AS Ross SA Khan IA ElSohly MA Potency trends of Δ^9^-THC and other cannabinoids in confiscated cannabis preparations from 1993 to 2008. J Forensic Sci 2010 55 1209 1710 10.1111/j.1556-4029.2010.01441.x 20487147

[B11] Gaoni Y Mechoulam R Isolation, structure, and partial synthesis of an active constituent of hashish. J Am Chem Soc 1964 86 1646 1647 10.1021/ja01062a046

[B12] Joy JE Watson SJ Benson JA (eds.) Marijuana and Medicine: Assessing the Science Base 1999 National Academies Press 25101425

[B13] Mechoulam R Plant cannabinoids: a neglected pharmacological treasure trove. Br J Pharmacol 2005 146 913 915 10.1038/sj.bjp.0706415 16205721 PMC1751232

[B14] DeLong GT Wolf CE Poklis A Lichtman AH Pharmacological evaluation of the natural constituent of *Cannabis sativa*, cannabichromene and its modulation by Δ^9^-tetrahydrocannabinol. Drug Alcohol Depend 2010 112 126 133 10.1016/j.drugalcdep.2010.05.019 20619971 PMC2967639

[B15] Izzo AA Borrelli F Capasso R Marzo V Di Mechoulam R Non-psychotropic plant cannabinoids: new therapeutic opportunities from an ancient herb. Trends Pharmacol Sci 2009 30 515 527 10.1016/j.tips.2009.07.006 19729208

[B16] Sirikantaramas S Morimoto S Shoyama Y Ishikawa Y Wada Y Shoyama Y Taura F The gene controlling marijuana psychoactivity: molecular cloning and heterologous expression of Δ^1^-tetrahydrocannabinolic acid synthase from *Cannabis sativa *L. J Biol Chem 2004 279 39767 39774 10.1074/jbc.M403693200 15190053

[B17] Taura F Sirikantaramas S Shoyama Y Yoshikai K Shoyama Y Morimoto S Cannabidiolic-acid synthase, the chemotype-determining enzyme in the fiber-type *Cannabis sativa*. FEBS Lett 2007 581 2929 2934 10.1016/j.febslet.2007.05.043 17544411

[B18] Taura F Tanaka S Taguchi C Fukamizu T Tanaka H Shoyama Y Morimoto S Characterization of olivetol synthase, a polyketide synthase putatively involved in cannabinoid biosynthetic pathway. FEBS Lett 2009 583 2061 2066 10.1016/j.febslet.2009.05.024 19454282

[B19] Potter D Clark P Brown M Potency of Δ^9^-THC and other cannabinoids in cannabis in England in 2005: Implications for psychoactivity and pharmacology. J Forensic Sci 2008 53 90 94 10.1111/j.1556-4029.2007.00603.x 18279244

[B20] Ming R Bendahmane A Renner SS Sex chromosomes in land plants. Ann Rev Plant Biol 2011 62 485 514 10.1146/annurev-arplant-042110-103914 21526970

[B21] Sakamoto K Akiyama Y Fukui K Kamada H Satoh S Characterization; genome sizes and morphology of sex chromosomes in hemp (*Cannabis sativa *L.) Cytologia 1998 63 459 464 10.1508/cytologia.63.459

[B22] NCBI database search October 12, 2011.

[B23] Marks MD Tian L Wenger JP Omburo SN Soto-Fuentes W He J Gang DR Weiblen GD Dixon RA Identification of candidate genes affecting Δ^9^-tetrahydrocannabinol biosynthesis in *Cannabis sativa*. J Exp Bot 2009 60 3715 2610 10.1093/jxb/erp210 19581347 PMC2736886

[B24] Rosenthal E The Big Book of Buds, Volume 3: More Marijuana Varieties from the World's Great Seed Breeders 2007

[B25] Li R Zhu H Ruan J Qian W Fang X Shi Z Li Y Li S Shan G Kristiansen K Li S Yang H Wang J Wang J De novo assembly of human genomes with massively parallel short read sequencing. Genome Research 2010 20 265 272 10.1101/gr.097261.109 20019144 PMC2813482

[B26] Simpson JT Wong K Jackman SD Schein JE Jones SJM Birol I ABySS: a parallel assembler for short read sequence data. Genome Research 2009 19 1117 1123 10.1101/gr.089532.108 19251739 PMC2694472

[B27] Grabherr MG Haas BJ Yassour M Levin JZ Thompson DA Amit I Adiconis X Fan L Raychowdhury R Zeng Q Chen Z Mauceli E Hacohen N Gnirke A Rhind N Palma F di Birren BW Nusbaum C Lindblad-Toh K Friedman N Regev A Full-length transcriptome assembly from RNA-Seq data without a reference genome. Nature Biotech 2011 29 644 652 10.1038/nbt.1883 PMC3571712 21572440

[B28] Mi H Lazareva-Ulitsky B Loo R Kejariwal A Vandergriff J Rabkin S Guo N Muruganujan A Doremieux O Campbell MJ Kitano H Thomas PD The PANTHER database of protein families, subfamilies, functions and pathways. Nucleic Acids Res 2005 33 D284 288 10.1093/nar/gki418 15608197 PMC540032

[B29] TAIR10 Genome Release. http://www.arabidopsis.org/

[B30] The Cannabis Genome Browser. http://genome.ccbr.utoronto.ca/

[B31] Su AI Cooke MP Ching KA Hakak Y Walker JR Wiltshire T Orth AP Vega RG Sapinoso LM Moqrich A Patapoutian A Hampton GM Schultz PG Hogenesch JB Large-scale analysis of the human and mouse transcriptomes. Proc Nat Acad Sci USA 2002 99 4465 4470 10.1073/pnas.012025199 11904358 PMC123671

[B32] Galbraith DW Birnbaum K Global studies of cell type-specific gene expression in plants. Ann Rev Plant Biol 2006 57 451 475 10.1146/annurev.arplant.57.032905.105302 16669770

[B33] Page JE Nagel J Romeo JT Biosynthesis of terpenophenolics in hop and cannabis. Recent Advances in Phytochemistry Volume 40: Integrative Plant Biochemistry 2006 Oxford: Elsevier 179 210

[B34] Phillips MA León P Boronat A Rodríguez-Concepción M The plastidial MEP pathway: unified nomenclature and resources. Trends Plant Sci 2008 13 619 623 10.1016/j.tplants.2008.09.003 18948055

[B35] Fellermeier M Eisenreich W Bacher A Zenk MH Biosynthesis of cannabinoids. Incorporation experiments with ^13^C-labeled glucoses. Eur J Biochem 2001 268 1596 1604 10.1046/j.1432-1327.2001.02030.x 11248677

[B36] Page JE Boubakir Z Aromatic prenyltransferase from cannabis 2011 PCT patent application WO/2011/017798

[B37] Turner JC Hemphill JK Mahlberg PG Quantitative determination of cannabinoids in individual glandular trichomes of *Cannabis sativa *L. (Cannabaceae). Am J Bot 1978 65 1103 1106 10.2307/2442328

[B38] Callaway JC Laakkonen TT Cultivation of Cannabis oil seed varieties in Finland. J Int Hemp Assoc 1996 3 32 34

[B39] Pollack JR Perou CM Alizadeh AA Eisen MB Pergamenschikov A Williams CF Jeffrey SS Botstein D Brown PO Genome-wide analysis of DNA copy-number changes using cDNA microarrays. Nat Genet 1999 23 41 46 10471496 10.1038/12640

[B40] Schneider K Kienow L Schmelzer E Colby T Bartsch M Miersch O Wasternack C Kombrink E Stuible H-P A new type of peroxisomal acyl-coenzyme A synthetase from *Arabidopsis thaliana *has the catalytic capacity to activate biosynthetic precursors of jasmonic acid. J Biol Chem 2005 280 13962 13972 10.1074/jbc.M413578200 15677481

[B41] Medicinal Genomics, LLC. http://www.medicinalgenomics.com/

[B42] Virovets VG Selection for non-psychoactive hemp varieties (*Cannabis sativa *L.) in the CIS (former USSR). J Int Hemp Assoc 1996 3 13 15

[B43] de Meijer EPM Bagatta M Carboni A Crucitti P Moliterni VMC Ranalli P Mandolino G The inheritance of chemical phenotype in *Cannabis sativa *L. Genetics 2003 163 335 346 12586720 10.1093/genetics/163.1.335 PMC1462421

[B44] de Meijer EPM Hammond K Micheler M The inheritance of chemical phenotype in *Cannabis sativa *L.(III): variation in cannabichromene proportion. Euphytica 2009 165 293 311 10.1007/s10681-008-9787-1

[B45] Morimoto S Komatsu K Taura F Shoyama Y Purification and characterization of cannabichromenic acid synthase from *Cannabis sativa*. Phytochemistry 1998 49 1525 1529 10.1016/S0031-9422(98)00278-7 9862135

[B46] Dittrich H Kutchan TM Molecular cloning, expression, and induction of berberine bridge enzyme, an enzyme essential to the formation of benzophenanthridine alkaloids in the response of plants to pathogenic attack. Proc Nat Acad Sci USA 1991 88 9969 9973 10.1073/pnas.88.22.9969 1946465 PMC52848

[B47] Datwyler SL Weiblen GD Genetic variation in hemp and marijuana (*Cannabis sativa *L.) according to amplified fragment length polymorphisms. J Forensic Sci 2006 51 371 351 10.1111/j.1556-4029.2006.00061.x 16566773

[B48] Faeti V Mandolino G Ranalli P Genetic diversity of *Cannabis sativa *germplasm based on RAPD markers. Plant Breeding 1996 115 367 370 10.1111/j.1439-0523.1996.tb00935.x

[B49] Ouyang S Buell CR The TIGR Plant Repeat Databases: a collective resource for the identification of repetitive sequences in plants. Nucleic Acids Res 2004 32 D360 363 10.1093/nar/gkh099 14681434 PMC308833

[B50] Stack SM Royer SM Shearer LA Chang SB Giovannoni JJ Westfall DH White RA Anderson LK Role of fluorescence in situ hybridization in sequencing the tomato genome. Cytogenet Genome Res 2009 124 339 350 10.1159/000218137 19556785

[B51] Russo EB Taming THC: potential cannabis synergy and phytocannabinoid-terpenoid entourage effects. British J Pharmacol 2011 163 1344 1364 10.1111/j.1476-5381.2011.01238.x PMC3165946 21749363

[B52] Maione S Piscitelli F Gatta L Vita D Petrocellis L De Palazzo E Novellis V de Marzo V Di Non-psychoactive cannabinoids modulate the descending pathway of antinociception in anaesthetized rats through several mechanisms of action. British J Pharmacol 2011 162 584 596 10.1111/j.1476-5381.2010.01063.x PMC3041249 20942863

[B53] Appendino G Gibbons S Giana A Pagani A Grassi G Stavri M Smith E Rahman MM Antibacterial cannabinoids from *Cannabis sativa*: a structure-activity study. J Nat Prod 2008 71 1427 1430 10.1021/np8002673 18681481

[B54] Wink M Plant breeding: importance of plant secondary metabolites for protection against pathogens and herbivores. Theor Appl Genet 1988 75 225 233 10.1007/BF00303957

[B55] Kliebenstein DJ Osbourn AE, Lanzotti V Use of secondary metabolite variation in crop improvement. Plant-Derived Natural Products: Synthesis, Function, and Application 2009 Springer 83 95

[B56] Doebley JF Gaut BS Smith BD The molecular genetics of crop domestication. Cell 2006 127 1309 1321 10.1016/j.cell.2006.12.006 17190597

[B57] Myles S Chia J-M Hurwitz B Simon C Zhong GY Buckler E Ware D Rapid genomic characterization of the genus *Vitis*. PloS One 2010 5 e8219 10.1371/journal.pone.0008219 20084295 PMC2805708

[B58] Myles S Boyko AR Owens CL Brown PJ Grassi F Aradhya MK Prins B Reynolds A Chia J-M Ware D Bustamante CD Buckler ES Genetic structure and domestication history of the grape. Proc Nat Acad of Sci USA 2011 108 3530 3535 10.1073/pnas.1009363108 21245334 PMC3048109

[B59] Landry BS Hubert N Etoh T Harada JJ Lincoln SE A genetic map for *Brassica napus *based on restriction fragment length polymorphisms detected with expressed DNA sequences. Genome 1991 34 543 552 10.1139/g91-084

[B60] Cheung W Champagne G Hubert N Landry B Comparison of the genetic maps of *Brassica napus *and *Brassica oleracea*. Theor Appl Genet 1997 94 569 582 10.1007/s001220050453

[B61] Meisel L Fonseca B Gonzaelz S Baeza-Yates R Cambiazo V Campos R Gonzalez M Orellana A Retamales J Silva H A rapid and efficient method for purifying high quality total RNA from peaches (*Prunus persica*) for functional genomics analyses. Biol Res 2005 38 83 88 15977413 10.4067/s0716-97602005000100010

[B62] Martin M Cutadapt removes adapter sequences from high-throughput sequencing reads. EMBnet J 2011 17 10 12

[B63] Langmead B Trapnell C Pop M Salzberg SL Ultrafast and memory-efficient alignment of short DNA sequences to the human genome. Genome Biol 2009 10 R25 10.1186/gb-2009-10-3-r25 19261174 PMC2690996

[B64] Miller JR Delcher AL Koren S Venter E Walenz BP Brownley A Johnson J Li K Mobarry C Sutton G Aggressive assembly of pyrosequencing reads with mates. Bioinformatics 2008 24 2818 2410 10.1093/bioinformatics/btn548 18952627 PMC2639302

[B65] Gish W States DJ Identification of protein coding regions by database similarity search. Nat Genet 1993 3 266 272 10.1038/ng0393-266 8485583

[B66] Kent WJ BLAT-the BLAST-like alignment tool. Genome Res 2002 12 656 664 11932250 10.1101/gr.229202 PMC187518

[B67] Li W Godzik A Cd-hit: a fast program for clustering and comparing large sets of protein or nucleotide sequences. Bioinformatics 2006 22 1658 1659 10.1093/bioinformatics/btl158 16731699

[B68] Huang X Madan A CAP3: A DNA sequence assembly program. Genome Res 1999 9 868 877 10.1101/gr.9.9.868 10508846 PMC310812

[B69] Li H Handsaker B Wysoker A Fennell T Ruan J Homer N Marth G Abecasis G Durbin R The Sequence Alignment/Map format and SAMtools. Bioinformatics 2009 25 2078 2079 10.1093/bioinformatics/btp352 19505943 PMC2723002

[B70] Loken C Gruner D Groer L Peltier R Bunn N Craig M Henriques T Dempsey J Yu C Chen J Dursi JL Chong J Northrup S Pinto J Knecht N Van Zon R SciNet: Lessons Learned from Building a Power-efficient Top-20 System and Data Centre. J Phys: Conf Ser 2010 256 012026

[B71] Tamura K Peterson D Peterson N Stecher G Nei M Kumar S MEGA5: Molecular evolutionary genetics analysis using maximum likelihood, evolutionary distance, and maximum parsimony methods. Mol Biol Evol 2011 28 2731 2739 10.1093/molbev/msr121 21546353 PMC3203626

